# Coronary catheterization via distal transradial access in patient with superficial radial artery: a case report

**DOI:** 10.1186/s12872-021-02444-1

**Published:** 2021-12-28

**Authors:** Yuan Fu, Lefeng Wang, Zhiyong Zhang, Kun Xia, Li Xu

**Affiliations:** Heart Center, Beijing Chaoyang Hospital, Capital Medical University, Beijing, China

**Keywords:** Radial artery, Anatomical variation, Superficial radial artery, Distal transradial access, Percutaneous coronary intervention

## Abstract

**Background:**

The routine radial artery (RA) puncture may fail when anatomical variation of the RA is encountered. Superficial radial artery (SRA) is one of the anatomic variants of the RA, with the incidence of about 1 to 1.5%. Recently, distal transradial access (dTRA) has emerged as a novel approach for coronary catheterization (CC), but performing CC through dTRA in patient with SRA has never been reported.

**Case presentation:**

A 57-year-old male was admitted to hospital due to intermittent chest pain for 4 days. He was diagnosed with unstable angina pectoris and planned to receive coronary angiography (CAG). Before the operation, the existence and course of SRA were confirmed by palpation and ultrasonography with color Doppler. We marked the puncture site under the guidance of ultrasonography and successfully performed CC through the dTRA during patient’s hospitalization.

**Conclusions:**

As far as we know, this is the first report that presents a case of SRA and percutaneous coronary intervention (PCI) treatment in which was successfully performed through dTRA. It is safe and feasible to perform CC via dTRA in case of SRA, and dTRA seems to be the preferred access.

## Introduction

The transradial access (TRA) is currently considered as the default option for coronary angiography (CAG) and percutaneous coronary intervention (PCI) because of the earlier mobilization, greater patient comfort, lower risk of vascular complications and major adverse cardiovascular events (MACE) [1, 2]. Radial artery (RA) puncture is usually performed based on blind palpation, however, routine RA puncture in the usual site may fail in case of RA anatomical variation [3, 4].

As one such variant of the RA, superficial radial artery (SRA) is a large branch vessel originates from the RA at the region 5 to 7 cm proximal from the distal wrist crease, and the incidence of SRA ranges from 1 to 1.5% [4, 5]. In recent years, distal TRA (dTRA) has emerged as a novel approach for coronary catheterization (CC), and a number of studies have demonstrated the safety and feasibility of dTRA for CAG and PCI [6, 7]. However, performing CAG and PCI via dTRA in case of SRA has never been reported before. We here present the first case for reference.

## Case presentation

**Fig. 1:**
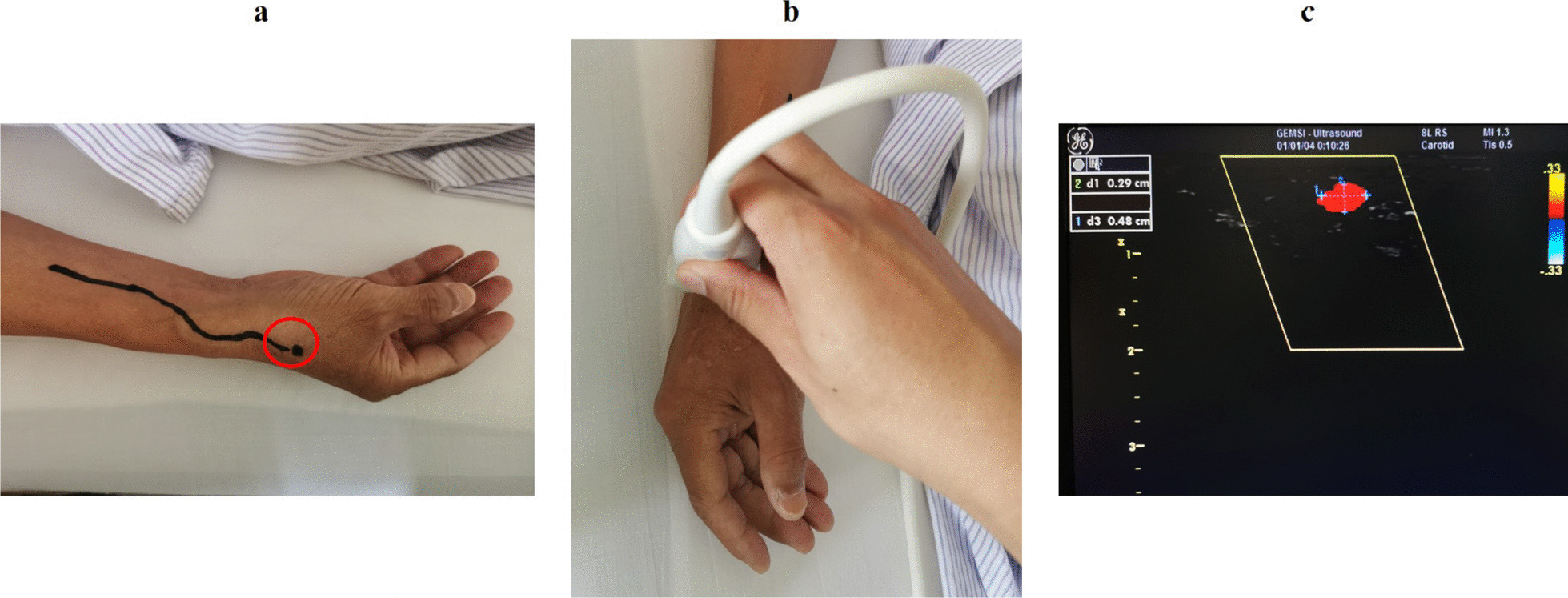
Identification of the superficial radial artery under the guidance of ultrasonography. **a** The course of the superficial radial artery (black line) and the puncture site of the distal radial artery (black dot) in the anatomic snuffbox (red circle) were marked. **b** The assessment of the distal radial artery condition. **c** The diameter of the distal radial artery lumen was 0.29 × 0.48 cm

A 57-year-old male was admitted to Beijing Chaoyang Hospital due to intermittent chest pain for 4 days. He had a history of hypertension for 5 years and smoking for about 30 years. The electrocardiogram (ECG) showed ST-segment depression (< 0.1 mV) in lead V2–V5. Except for the level of low-density lipoprotein cholesterol (LDL-C, 3.58 mmol/L), other laboratory exams were all unremarkable. He was diagnosed with unstable angina pectoris and planned to receive CAG. Before the selective CAG, the pulse of right RA was routinely examined. Interestingly, the RA was weak but pulsatile at the routine puncture site in the anterior region of the forearm, and the course of the RA could not be palpated clearly. Meanwhile, the pulsation of the RA was strong at the dorsal lateral side of the wrist, approximately 2 to 3 cm cephalad to the anatomic snuffbox (AS) and the course of the vessel was palpable. Ultrasonography with color Doppler was then performed and the existence and course of SRA was confirmed. It went down subcutaneously to the dorsal lateral side of the forearm, and finally into the AS, which was consistent with the pulsation before. We marked the course of the SRA and the puncture site of the distal RA under the guidance of ultrasonography (Fig. 1).

**Fig. 2:**
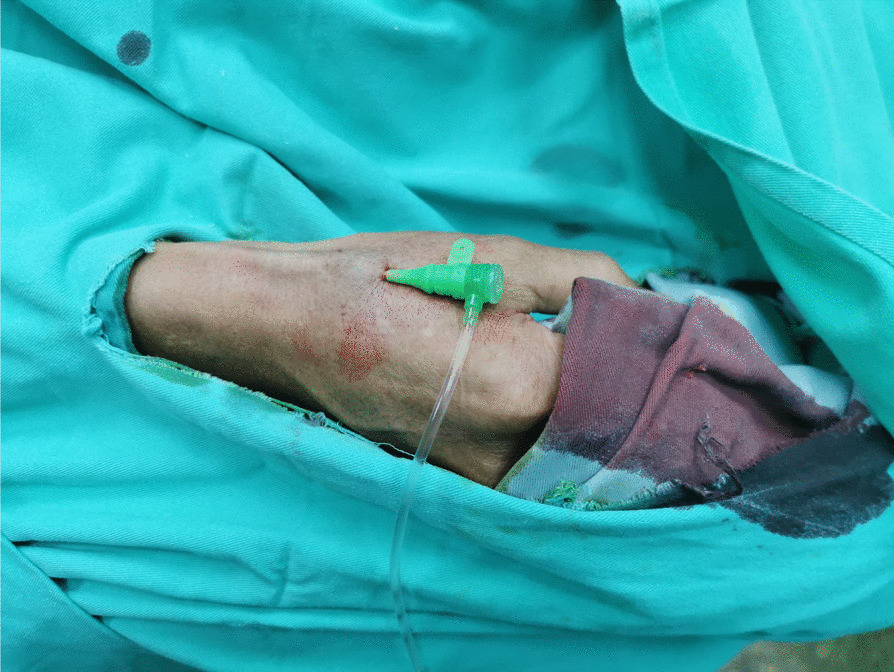
Puncture and sheath placement in the distal radial artery

**Fig. 3:**
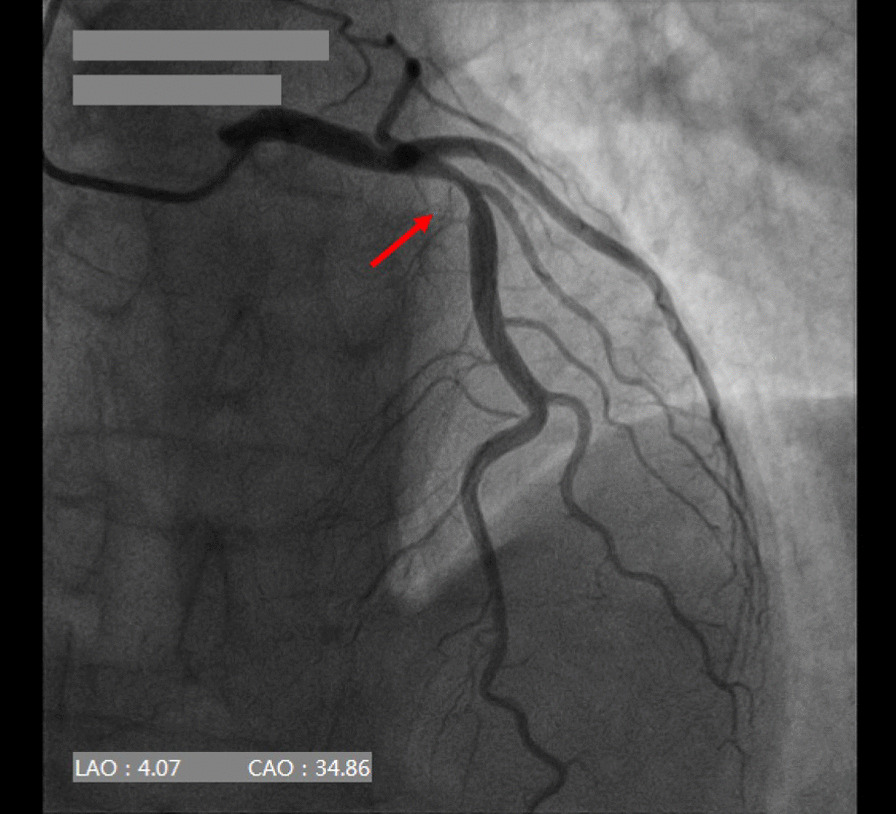
Coronary angiography revealed a severe stenosis in the left anterior descending coronary artery

**Fig. 4:**
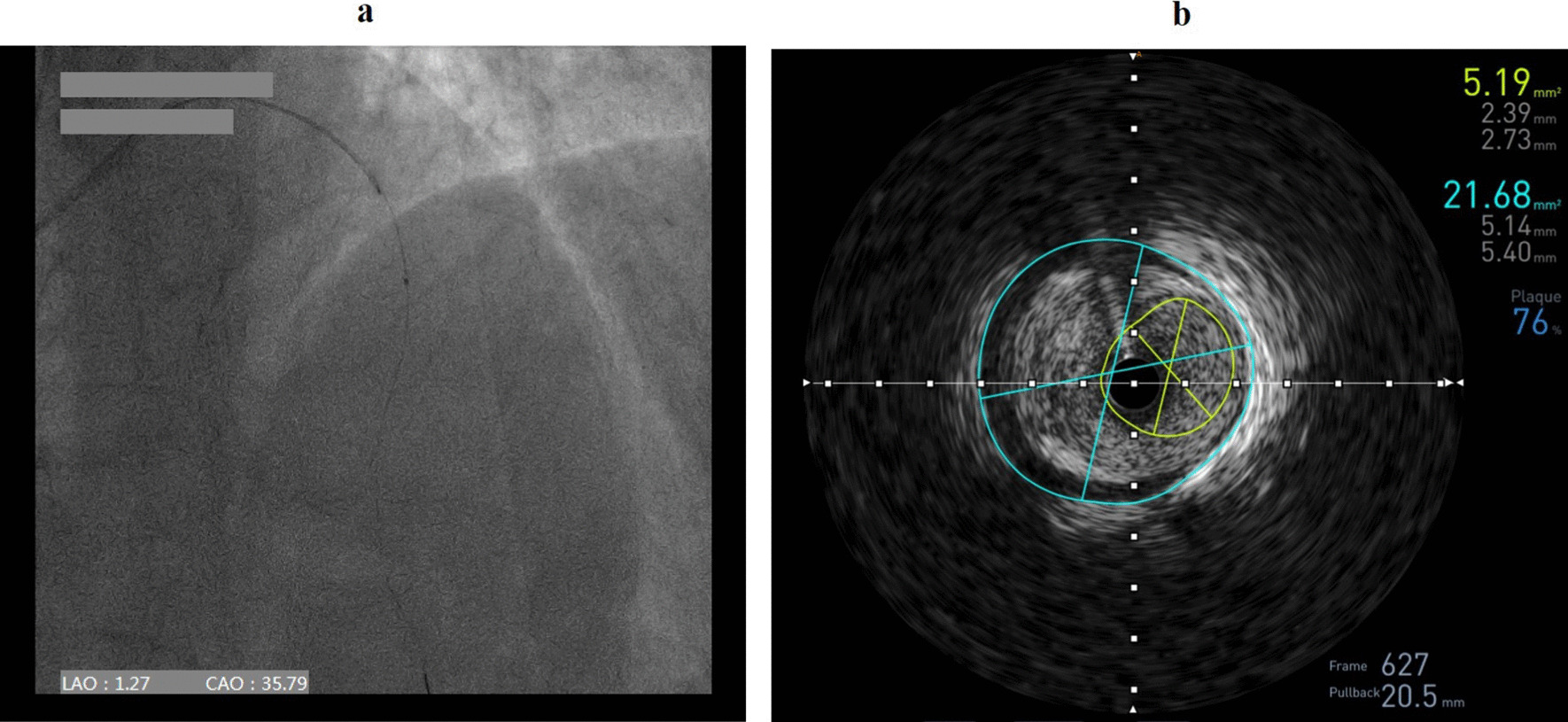
Intravenous ultrasound images. **a** Intravenous ultrasound (IVUS) images were acquired by automated mechanical pullback devices with a continuous pull back speed of 0.5 mm/s. **b** The lumen area of the LAD lesion was 5.19 mm^2^ and the plaque load was 76%

**Fig. 5:**
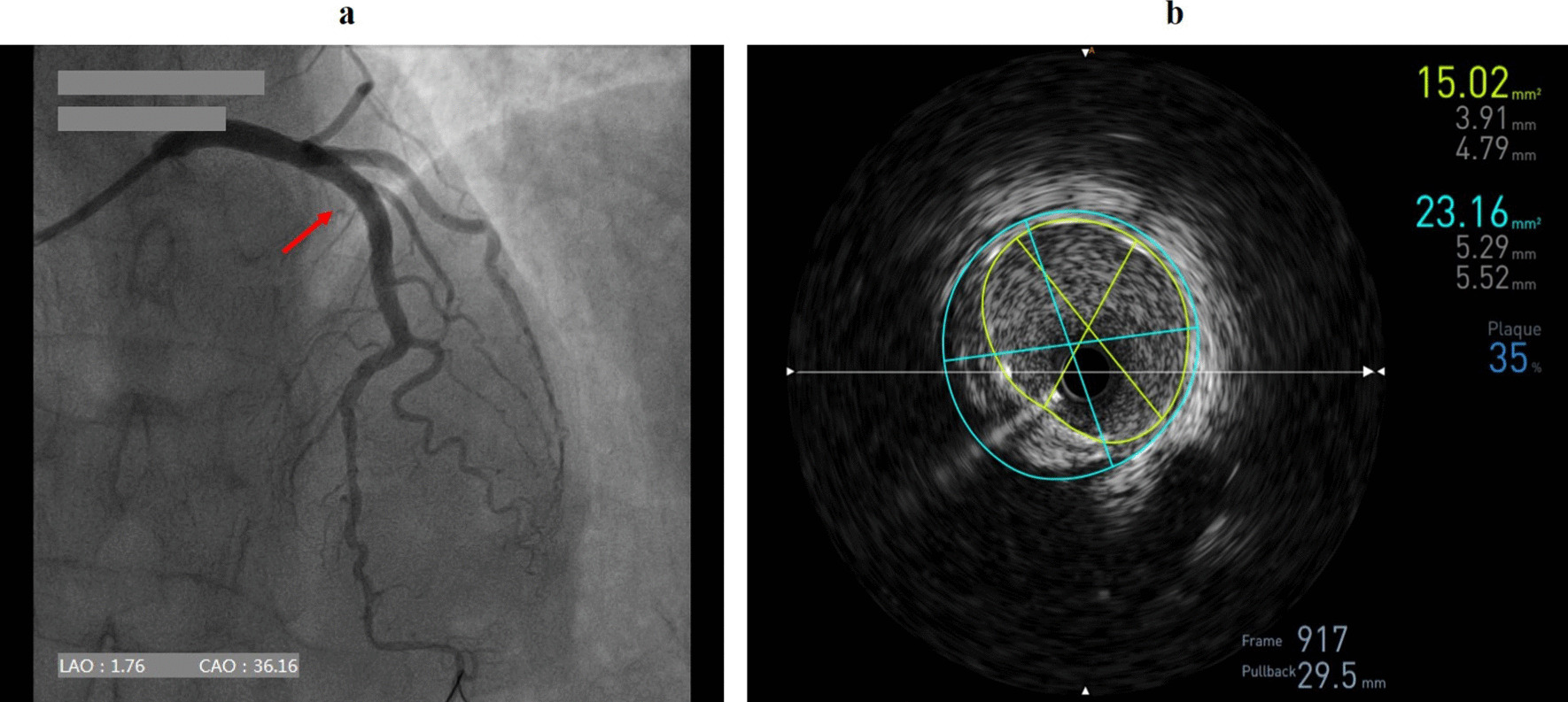
Post-procedure images. **a** The final angiographic appearance of the left anterior descending coronary artery (LAD). **b** The lumen area of the LAD after stent implantation was 15.02 mm^2^ and the plaque load was reduced to 35%

**Fig. 6:**
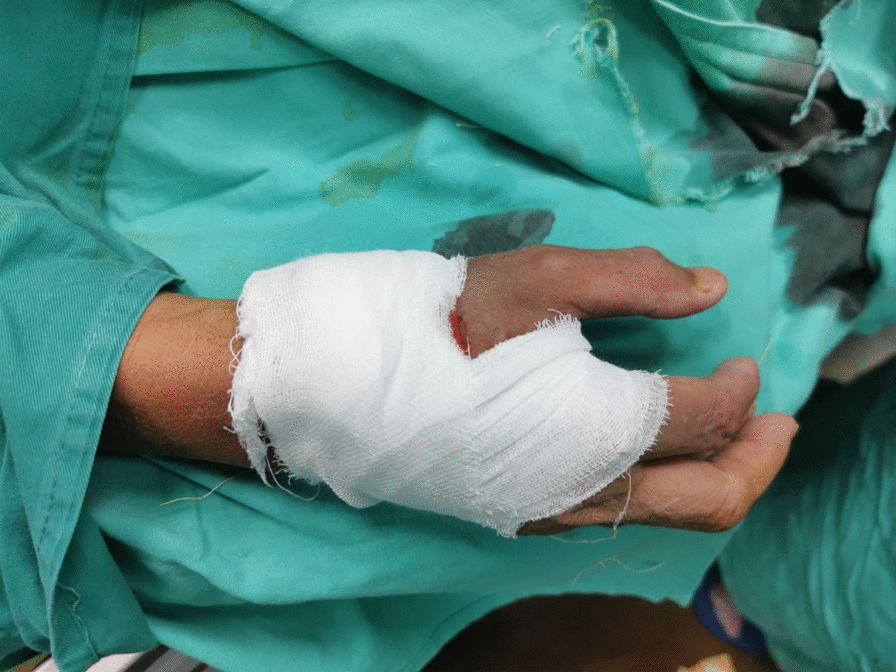
Hemostasis was performed by compression with gauze and bandage

Puncture of the distal RA in the AS was performed using a Terumo angiocatheter needle and a 6 Fr Terumo introducer sheath was then inserted into the vessel (Fig. 2). CAG revealed a severe stenosis (80–90%) in the proximal to middle segment of the left anterior descending coronary artery (LAD, Fig. 3). In order to evaluate the lesion more accurately, intravenous ultrasound (IVUS, Boston Scientific, USA) was performed. After intracoronary injection of 200 mg of nitroglycerine, IVUS images were acquired by automated mechanical pullback devices with a continuous pull back speed of 0.5 mm/s (Fig. 4a). The results of the IVUS showed that the lumen area of the LAD lesion was 5.19 mm^2^ and the plaque load was 76% (Fig. 4b). A 4.0 × 30 mm drug-eluting stent (DES, Resolute Integrity, Medtronic, USA) was then implanted into proximal to middle segment of the LAD. After the stent was postdilated at nominal pressure with a 4.5 × 15 mm and a 5.0 × 15 mm noncompliant balloon (NCSprinter, Medtronic, USA), the final angiographic appearance and the IVUS images were satisfactory (Fig. 5). The 6Fr Terumo sheath was removed from the distal RA immediately after the procedure and the puncture site was compressed with bandage and gauze for about 6 h (Fig. 6). The total time of the procedure was 65 min, with the arterial puncture time (time from puncture of the distal RA in the AS to sheath insertion) was 3 min. The total amount of contrast was 110 ml. The patient was discharged the next day after the procedure, the distal RA was palpable in the puncture site and the course of SRA was also palpable. One month after the procedure, the patient was followed up in the outpatient department, the pulsation of the distal RA in the AS and the course of SRA were both palpable.

## Discussion and conclusions

TRA is recommended as the routine approach for CAG and PCI nowadays [1, 8]. PCI via TRA has lower risk of vascular complications, all-cause mortality and MACE, compared with PCI via transfemoral access (TFA) [2, 9]. Recently, dTRA has emerged as a novel approach for CAG and PCI. It was first introduced by Kiemeneij in 2017 and since then, the feasibility and safety of CC through dTRA have been proved by many studies [6, 7, 10]. Compared with TRA, this technique has advantages in faster hemostasis, lower risk of radial artery occlusion (RAO) and recanalizing RA stenosis or RAO [7, 11]. However, none of these previous studies reported the safety and feasibility of CAG and PCI via dTRA in case of RA anatomical variation, such as SRA.

SRA is a large branch vessel arises from the RA in the distal fourth of the forearm, 5 to 7 cm proximal from the distal wrist crease, with an incidence between 1 and 1.5% [3, 5]. SRA extends subcutaneously on the radial side of the forearm, over the tendon of the brachioradialis muscle, passes over the tendon of the extensor pollicis brevis into the AS and forms the deep palmar arch of the hand with the ulnar artery [3–5]. It becomes difficult to puncture RA in the traditional puncture site of the TRA in case of SRA. For instance, Hudcova et al. reported a difficult arterial catheter placement case with SRA, and eventually cannulation of the RA in the AS [3]. SRA was not identified before surgery in the case report of Uchino et al., and they failed to place arterial catheter in the usual RA puncture site after multiple attempts [4]. In our case report, the pulsation of the SRA was visible and could be detected by palpation. However, there was also a weak pulsation of the RA at the usual puncture site in the anterior region of the forearm. The RA, after the SRA split, ran a normal course deep into the muscles of the forearm might cause this situation, similar to the case report of Uchino et al. Ultrasonography with color Doppler was then performed, the course of the RA could not be observed at the routine puncture site but the existence and course of the SRA was confirmed. CAG and PCI through dTRA has many advantages and the SRA just ran down into the AS in our case, we therefore selected distal RA as the CC approach. After the assessment of the artery condition of the dTRA puncture site (Fig. 1c), we successfully performed CC via dTRA with no vascular complications occurred.

To the best of our knowledge, this is the first report that presents a case of SRA and CC in which was successfully performed via dTRA. According to the anatomical morphology of the SRA and the benefits of CC via dTRA, we think dTRA may be the best access among SRA patients with CC plan. Because SRA extends subcutaneously on the radial side of the forearm and there is no forearm muscle around SRA for protection, great care should be taken when inserting the arterial sheath to prevent vascular complications. This might be the potential disadvantage of the current SRA approach. However, the safety and feasibility of this technique should be verified by long-term follow up and in a large-scale population.

## Data Availability

All relevant information is contained within the present manuscript.

## Abbreviations

RA: Radial artery
SRA: Superficial radial artery
dTRA: Distal transradial access
CC: Coronary catheterization
CAG: Coronary angiography
PCI: Percutaneous coronary intervention
TRA: Transradial access
MACE: Major adverse cardiovascular events
ECG: Electrocardiogram
LDL-C: Low-density lipoprotein cholesterol
AS: Anatomic snuffbox
LAD: Left anterior descending coronary artery
IVUS: Intravenous ultrasound
TFA: Transfemoral access
RAO: Radial artery occlusion
